# Surgical management of large adrenal tumors: impact of different laparoscopic approaches and resection methods on perioperative and long-term outcomes

**DOI:** 10.1186/s12894-018-0349-0

**Published:** 2018-05-08

**Authors:** Wei Chen, Yong Liang, Wei Lin, Guang-Qing Fu, Zhi-Wei Ma

**Affiliations:** 1 Department of Urology, Zigong No.4 People’s Hospital, Sichuan, 643000 China; 2 Department of Urology, Sichuan Academy of Medical Sciences & Sichuan Provincial People’s Hospital, No.32 West Second Section First Ring Road, Chengdu, 641000 Sichuan China

**Keywords:** Retroperitoneal laparoscopic adrenalectomy, Minimally invasive surgery, Adrenal tumor

## Abstract

**Background:**

The indication of retroperitoneal laparoscopic adrenalectomy (RLA) was extended with the retroperitoneal approach and has been wildly accepted and technologically matured. However, the management of large adrenal tumors via this approach still remains controversial. The aim of this study was to perform a comprehensive analysis on the minimally invasive surgical management of larger adrenal tumors.

**Methods:**

A total of 78 patients with large adrenal tumors (> 5 cm) and 97 patients with smaller adrenal tumors (< 5 cm) were enrolled in this study. The patient characteristics were preferentially analyzed. The intra-operative and postoperative indicators were compared between those who underwent RLA and those who underwent transperitoneal laparoscopic adrenalectomy (TLA); the intra-operative and postoperative indicators were also compared between the large tumor group and smaller tumor group of those who underwent RLA. Furthermore, the analyses of partial RLA were focused on the perioperative indicators and follow-up results.

**Results:**

RLA was superior to TLA in terms of operation time (98.71 ± 32.30 min vs. 124.36 ± 34.62 min, respectively, *P* = 0.001), hospitalization duration (7.43 ± 2.82 days vs. 8.91 ± 3.40 days, respectively, *P* = 0.04), duration of drain (4.83 ± 0.37 days vs. 3.94 ± 2.21 days, respectively, *P* = 0.02), first oral intake (2.82 ± 0.71 days vs. 1.90 ± 0.83 days, respectively, *P* < 0.001) and time to ambulation (3.89 ± 1.64 days vs. 2.61 ± 1.42 days, respectively, *P* < 0.001). Further analyses of the RLA patients demonstrated that the larger tumor (> 5 cm) group showed superior results for the intraoperative indicators than the smaller tumor (< 5 cm) group (*P* < 0.05), while the results for the postoperative indicators between the two tumor size groups were similar (*P* > 0.05). Data confirmed that the partial resection method was superior to the total resection method from the perspective of the hormone supplement (0% vs. 48.15%, *P* = 0.002). The 2-year recurrence-free rates were 92.60 and 92.86% for the total and partial RLA resection methods, respectively (*P* = 0.97). The partial RLA resection method had a similar complete remission rate as the total RLA resection method (96.30% vs. 100%, respectively, *P* = 0.47).

**Conclusion:**

Both RLA and TLA seem to provide similar effects for the surgical management of large adrenal tumors. However, partial RLA resection should be considered for the management of benign tumors to reduce the hormone supplement.

**Electronic supplementary material:**

The online version of this article (10.1186/s12894-018-0349-0) contains supplementary material, which is available to authorized users.

## Background

Laparoscopic adrenalectomy, which includes both the retroperitoneal and transperitoneal approaches, has been regarded as the gold standard for the management of adrenal tumors. In a previous study, we demonstrated that the retroperitoneal approach was superior to the transperitoneal method in terms of the perioperative indicators [1]. However, it is known that the tumor diameter is an important factor in the surgical management of adrenal tumors using the retroperitoneal laparoscopic technique. Satisfactory clinical results of lateral retroperitoneal laparoscopic adrenalectomy (RLA) for small adrenal tumors have been reported [2–4]. Scholars have also reported that the largest adrenal benign tumor managed by the transperitoneal approach was 15 cm in diameter, and the largest adrenal benign tumor managed by the retroperitoneal method was 6 cm in diameter. Since the retroperitoneal approach has been wildly accepted and technologically matured, its indication has been extended. However, the management of large adrenal tumors, which are often defined as tumors larger than 5 cm, via this approach still remains controversial. There were approximately 800 cases of adrenal tumors that underwent operation in Sichuan Provincial People’s Hospital and Zigong Fourth People’s Hospital during 2011–2015. The percentage of large adrenal tumors was approximately 9.7%. The aim of this study was to perform a comprehensive analysis of the surgical management of larger adrenal tumors based on patient information from the databases of the abovementioned hospitals.

## Methods

The present study was designed as an observational study and approved by the hospital ethics committee of Zigong Fourth People’s Hospital and Sichuan Provincial People’s Hospital. All of the patients gave informed consent to participate in the study before the operation. A prospectively maintained urology database in the Department of Urology at Zigong Fourth People’s Hospital and Sichuan Provincial People’s Hospital was retrospectively reviewed to collect data on the large diameter complicated adrenal tumor patients who underwent an RLA or transperitoneal laparoscopic adrenalectomy (TLA) procedure. The present study adheres to the STROBE reporting guidelines (Additional file 1).

The inclusion criteria for our study were as follows: (i) the patient was confirmed as having an adrenal tumor by imaging examination and underwent minimally invasive surgery treatment, (ii) the baseline indicators and perioperative parameters were completely recorded, and (iii) a large adrenal tumor was defined as an adrenal tumor with a diameter larger than 5 cm. The exclusion criteria were as follows: (i) there were no measurable data reported, (ii) the tumor was operated on via open surgery, and (iii) a hand-assisted laparoscopic method was adopted during the surgery. From January 2011 to June 2015, there were 78 patients with an adrenal tumor larger than 5 cm. Of these 78 patients, 41 (26 males and 15 females) patients underwent RLA, and the other 37 (23 males and 14 females) patients underwent the TLA procedure. All of the patients received a complete laboratory examination, which included 17-keto-steroid, 17-hydroxycorticosteroids, vanilmandelic acid, aldosterone/renin ratio, aldosterone, renin and plasma metanephrines measurements. Image evaluations, such as computed tomography, were used to determine the localization and diameter of the adrenal mass. All of the patients were provided operative informed consent, which mentioned the benefits and potential risks of the proposed operation method. We also informed all of the patients that open conversion might occur if any difficulties were encountered during the operation.

### Surgical technique

The patients were divided into the RLA group and TLA group according to the approach of the laparoscopic technique that they underwent. Subgroups were formed in the RLA group based on the diameter or resection method used.

**Fig. 1:**
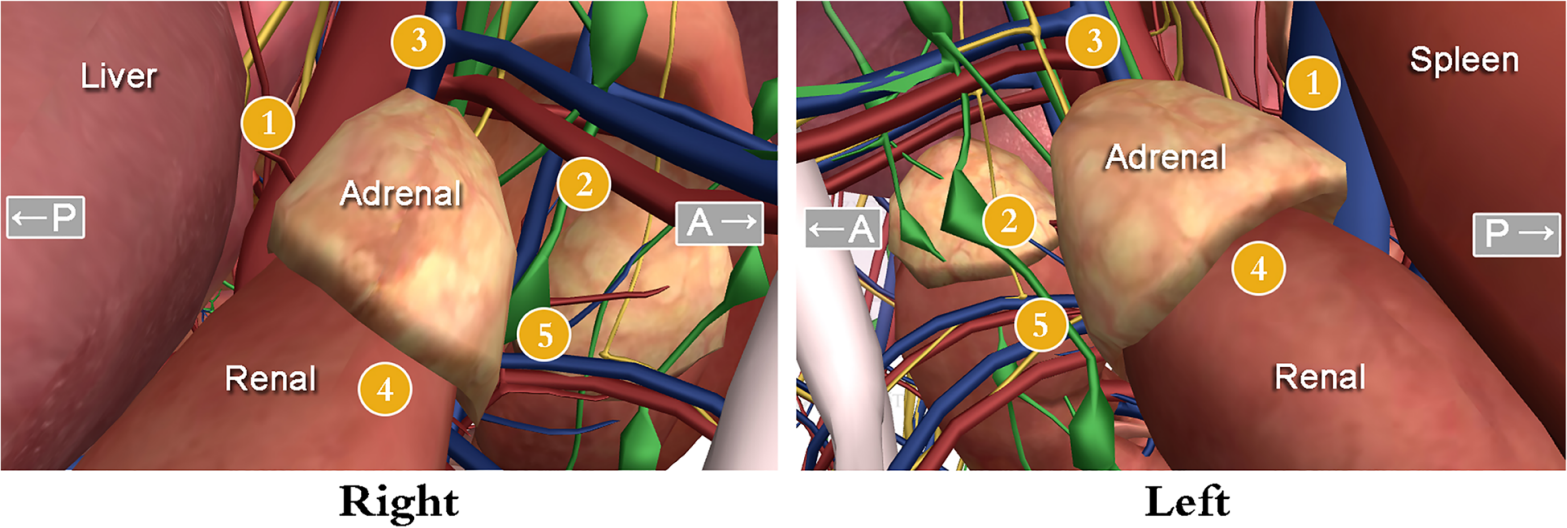
The steps used in the operation for the management of the right and left adrenalectomies. A: anterior; P: posterior

A lateral position was selected for these patients. A 30-degree laparoscope was used as an observation mirror though a 10-mm trocar, and the other two trocars were located in the anterior axillary line and posterior axillary line of the subcostal space at a diameter of 5 mm or 10 mm, respectively. The operation sequence was performed according to the steps shown in Fig. 1. Ultrasonic shears were used to divided and identify the edge of the adrenal gland. The central adrenal vein was divided between hem-o-lok clips. After the adrenal gland was completely dissected, a homemade bag was used to dress up the adrenal gland via a 10-mm trocar placed into the peritoneal cavity. The mass was cut and examined on the operation table to ensure the involvement of the entire tumor. After the adrenal area was exposed, special attention was given to the evaluation of the tumor location and determination of whether there was periadrenal involvement. The TLA method was administered as it is referred to in the paper reported by Mohammadi-Fallah et al. [5].

In both the RLA and TLA groups, the main principle of the procedure was a resection with as much peri-adrenal tissue as possible to ensure that the highest negative margin rate in malignant tumors was obtained. A drainage tube was routinely placed before the operation completed. All of the procedures were performed by an experienced senior laparoscopic surgeon to reduce the selective bias.

### Postoperative management

We routinely put the patients in the post-anesthesia care unit until their blood pressure and heart rate were stable and they regained consciousness after the surgery. Patients with significant bleeding or an unstable blood pressure would be transferred to the intensive care unit (ICU). After approximately 30 min of postoperative monitoring in the post-anesthesia care unit, the patients were transferred to the urology department for a half day to 1 day for electrocardiograph monitoring. A fasting period of 24 h to 2 days was implemented to help the bowel function recovery of the patients. During this period, postoperative intravenous fluid supplementation was used in all of the patients. Postoperative hypotension, which might have been caused by bleeding or an inadequate intravenous fluid supplementation during the intraoperative or postoperative period, was corrected in a timely manner. Fortunately, none of the patients required this management. The patients were discharged after an adequate pain control was achieved and after the drainage tube was extubated. In addition, patients could receive a regular diet and normal ambulation based on the requirement of the condition for achieving a discharge standard.

### Outcome definition

The basic characteristics of the patients were preferentially analyzed to assess the balance and comparability between the two groups. These characteristics included the mean age, gender, body mass index (BMI), tumor size, tumor side, previous abdominal surgery, resection method and pathology type of the patients.

To evaluate the efficacies of the RLA and TLA procedures, both the intra-operative terms and postoperative terms were required. The intra-operative terms included the operation time (OT), estimated blood loss (EBL), number of complications, and number of ICU conversions. The postoperative terms included hospitalization, duration of the draining, hormone supplementation, the time to first oral intake and the time to first ambulation. The follow-up indicators included the two-year recurrence-free rate (RFR) and complete remission (CR). RFR was defined as the detection of no new tumors and was based on clinical and imagology evidence. CR was defined as a complete disappearance of the manifestations of disease, which included hypertension, central obesity, osphyalgia, etc.

### Statistical analysis

All of the continuous data were expressed as the mean ± standard deviation, while the discontinuous data were presented as the percentage. The statistical analyses were performed using SPSS version 20.0 (SPSS Inc., Chicago, IL). Two independent-sample t tests were used to compare the continuous variables between the groups, and Chi-square tests were used to compare the discontinuous variables between the two groups. Curve estimations were used to explore the correlation between the tumor diameter and other potential factors. The estimation model included linear, logarithmic, inverse, quadratic, cubic, compound, power, s curve, growth curve and exponential analyses. *P* < 0.05 was used to indicate statistically significant differences between the groups.

## Results

### Patient characteristics

During January 2011 and June 2015, a total of 78 adrenalectomies were performed at Zigong Fourth People’s Hospital and Sichuan Provincial People’s Hospital. Of these procedures, the RLA approach was used in 41 patients (27 with a total excision and 14 with a partial excision), and the TLA approach was used in 37 patients (17 with a total excision and 20 with a partial excision). The groups consisted of 49 (62.82%) male patients and 29 (37.18%) female patients who had an age range of 21–67 years (mean age: 46.59 ± 35.60 years). The mean tumor diameter was 5.78 ± 1.29 cm, and there were no statistically significant differences between the groups (*P* = 0.86). The tumor localization and peritoneal invasion of each patient was previously evaluated by a computed tomography scan. There were 57 (69.23%) left-side and 3 (2.99%) bilateral-sided surgeries. Of the total patient population, 38 patients (48.72%) had the diagnosis of aldosterone-producing adenoma or adrenocortical hyperplasia, while 8 patients (10.26%) had the diagnosis of pheochromocytoma. There were 4 patients (5.13%) with hematoma and an adrenal cyst. In the patient population, there were 2 patients with adrenocortical carcinoma who received LRA and 4 patients with ACC who received TLA. Total adrenal resection was performed in 44 of the patients (56.41%). All of the basic characteristics showed no significant differences between the two groups (*P* > 0.05). The disease and patient characteristics are shown in Table 1.

**Table 1:** A comparison of the basic characteristics between the two groups

	Overall	RLA	TLA	*P* value
No. of patients	78	41	37	
Mean age (yrs)	46.59 ± 35.60	44.41 ± 38.90	47.92 ± 29.61	0.65
BMI (kg/m^2^)	23.58 ± 2.07	23.52 ± 2.46	23.60 ± 2.92	0.89
Tumor size (cm)	5.78 ± 1.29	5.81 ± 1.17	5.76 ± 1.34	0.86
Gender				0.90
Male	49	26	23	
Female	29	15	14	
Localization				0.79
Left	54	29	25	
Right	21	11	10	
Bilateral	3	1	2	
Previous abdominal surgery	3	1	2	0.49
Resection				0.07
Total	44	27	17	
Partial	34	14	20	
Pathology				0.47
Adrenocortical hyperplasia	18	10	8	
Aldosterone-producing adenoma	38	18	20	
Adrenal medullary hyperplasia	3	1	2	
Adrenal myelolipoma	1	1	0	
Adrenocortical carcinoma	6	2	4	
Adrenal cyst	3	3	0	
Hematoma	1	1	0	
Pheochromocytoma	8	5	3	

*BMI* body mass index, *RLA* retroperitoneal laparoscopic adrenalectomy, *TLA* transperitoneal laparoscopic adrenalectomy

### Retroperitoneal procedure versus Transperitoneal procedure

There were no significant differences in numbers of intra-operative estimated blood loss, conversions to the ICU and postoperative complications between the RLA and TLA groups (P > 0.05). The RLA group had a shorter operation time (98.71 ± 32.30 min vs. 124.36 ± 34.62 min, *P* = 0.001) and shorter hospitalization duration (7.43 ± 2.82 days vs. 8.91 ± 3.40 days, *P* = 0.04) compared to the TLA group. The TLA group had a longer duration of draining (4.83 ± 0.37 days vs. 3.94 ± 2.21 days, *P* = 0.02), longer time to first oral intake (2.82 ± 0.71 days vs. 1.90 ± 0.83 days, *P* < 0.001) and longer time to ambulation (3.89 ± 1.64 days vs. 2.61 ± 1.42 days, P < 0.001) than the RLA group (Table 2).

**Table 2:** A comparison of the operative and postoperative outcomes between RLA and TLA groups

	Overall	RLA	TLA	*P* value
No. of patients	78	41	37	
Convert to ICU	5	3	2	0.73
Complication	1	0	1	0.29
Operation time (min)	103.96 ± 32.09	98.71 ± 32.30	124.36 ± 34.62	0.001
Estimated blood loss (cc)	33.72 ± 21.34	31.93 ± 20.01	36.34 ± 19.83	0.33
Hospitalization (d)	8.09 ± 2.67	7.43 ± 2.82	8.91 ± 3.40	0.04
Duration of drain (d)	4.18 ± 2.92	3.94 ± 2.21	4.83 ± 0.37	0.02
First oral intake (d)	2.31 ± 0.75	1.90 ± 0.83	2.82 ± 0.71	< 0.001
Time to ambulation (d)	2.99 ± 1.68	2.61 ± 1.42	3.89 ± 1.64	< 0.001

*ICU* intensive care unit, *RLA* retroperitoneal laparoscopic adrenalectomy, *TLA* transperitoneal laparoscopic adrenalectomy

### Retroperitoneal approach for the management of tumors larger than 5 cm versus tumors smaller than 5 cm

During the same period of this study, there were 97 patients with a tumor of a diameter smaller than 5 cm who received retroperitoneal adrenal surgeries. Comparing between the larger tumor and smaller tumor groups, there were no significant differences in the number of conversions to the ICU, number of postoperative complications, time to first oral intake and time to ambulation. However, a longer operation time and higher intra-operative estimated blood loss were detected in the larger tumor group compared to the smaller tumor group (OT: 98.71 ± 32.30 min vs. 42.63 ± 18.51 min, *P* < 0.001; EBL: 31.93 ± 20.01 cm^3^ vs. 10.29 ± 6.04 cm^3^, *P* < < 0.001). Moreover, the larger tumor group experienced a longer hospitalization duration (7.43 ± 2.82 days vs. 2.07 ± 0.36 days, *P* < 0.001) and longer duration of draining (3.94 ± 2.21 days vs. 0.98 ± 0.07 days, *P* < 0.001) compared to the smaller tumor group (Table 3).

**Table 3:** A comparison of the perioperative indicators between the patients in the larger adrenal tumor and smaller adrenal tumor groups who underwent RLA

	Overall	> 5 cm	< 5 cm	*P* value
No. of patients	138	41	97	
Convert to ICU	3	3	2	0.13
Complication	1	0	1	0.52
Operation time	63.28 ± 28.91	98.71 ± 32.30	42.63 ± 18.51	< 0.001
Estimated blood loss	19.61 ± 12.50	31.93 ± 20.01	10.29 ± 6.04	< 0.001
Hospitalization	4.63 ± 1.95	7.43 ± 2.82	2.07 ± 0.36	< 0.001
Duration of drain	1.84 ± 1.52	3.94 ± 2.21	0.98 ± 0.07	< 0.001
First oral intake	1.16 ± 0.58	1.90 ± 0.83	1.72 ± 0.31	0.07
Time to ambulation	2.03 ± 1.07	2.61 ± 1.42	2.32 ± 0.48	0.08

*ICU* intensive care unit

### Retroperitoneoscopic total resection versus retroperitoneoscopic partial resection in large adrenal tumors

According to the tumor characteristics, a total of 14 of the 41 patients (34.15%) received partial adrenalectomy via the retroperitoneoscopic procedure. All of the 14 patients had benign tumors, which was confirmed by a careful postoperative pathological examination. The operation time for partial resection was 102.68 ± 30.92 min, whereas the operation time for total resection was 79.64 ± 28.39 min (*P* = 0.02). Partial resection was associated with a higher EBL than was total resection (45.19 ± 18.63 cm^3^ vs. 28.60 ± 21.75 cm^3^, P = 0.02). However, there were zero hormone supplements in the partial resection group, while this number in the total patient population was 13. The partial resection method was superior to the total resection method from the perspective of hormone supplementation (0% vs. 48.15%, *P* = 0.002) (Table 4).

**Table 4:** A comparison of the perioperative indicators between the different resection methods use in the RLA procedure for adrenal tumors with a diameter larger than 5 cm

	Overall	Total Resection	Partial Resection	*P* value
No. of patients	41	27	14	
Conversions to ICU	3	3	0	0.20
Hormone supplements	13	13	0	0.002
Tumor size (cm)	5.81 ± 1.17	5.92 ± 1.54	5.63 ± 1.08	0.49
Operation time	98.71 ± 32.30	79.64 ± 28.39	102.68 ± 30.92	0.02
Estimated blood loss	31.93 ± 20.01	28.60 ± 21.75	45.19 ± 18.63	0.02
Hospitalization	7.43 ± 2.82	7.05 ± 0.69	7.68 ± 1.52	0.07
Duration of draining	3.94 ± 2.21	3.60 ± 2.07	4.53 ± 2.78	0.23
First oral intake	1.90 ± 0.83	1.58 ± 0.61	2.06 ± 0.97	0.06
Time to ambulation	2.61 ± 1.42	2.14 ± 1.07	2.81 ± 0.96	0.05

### Follow-up results of retroperitoneal laparoscopic adrenalectomy

The 2-year RFR was 92.60% for the total resection RLA and was 92.86% for the partial resection RLA (*P* = 0.97). The rate of CR in the partial resection RLA group was similar to that of the total resection RLA group (26 (96.30%) vs. 14 (100%), *P* = 0.47).

## Discussion

Adrenal surgery is a type of high-risk operation that has been used in urology for a long time. In the past, even adrenal tumors with a small diameter needed a large incision and high position in the operation due to the special location of the adrenal gland [6]. This method not only causes great trauma to the patient but also significantly increases the numbers of pleural lesions and surgical complications. Most importantly, adrenal vasculature can only be ligated through blind separation and hand feeling because most adrenal tumors could show difficulties, thereby causing the probability of tissue injury and hemorrhage [7]. Laparoscopic adrenal surgery has gained widespread popularity, and this procedure could own a more fine and convenient operation that could clearly separate the important vessels under direct vision. This technique can significantly decrease the number of postoperative complications [8].

There are many methods of minimally invasive surgeries in the management of adrenal tumors. According to the surgical approach used, the methods can be divided into transperitoneal and retroperitoneal laparoscopy adrenalectomies [1]. Advantages and disadvantages have been reported for transperitoneal and retroperitoneal adrenalectomies. The transperitoneal approach benefits from more visibility and a larger working space, as well as involving the most familiar anatomy for surgeons. Previous opinions show that TLA is better than RLA in the treatment of large adrenal tumors (> 5 cm). However, TLA disturbs intra-abdominal structures and organs, which produces a high risk for organ or vascular injury. The complications of TLA also include a prolonged ileus and the risk for adhesion formation. In patients who received previous abdominal surgery, TLA is especially difficult to perform. While RLA owns obvious advantages, it has a more direct route and cannot interfere with the intra-abdominal organs. In our previous research, we identified that the operative time of RLA is shorter than that of TLA [1]. With the development of minimally invasive surgery, scholars constantly show that large adrenal tumors also benefit from the RLA approach.

Different positions, such as lateral, posterior or anterior, could be considered by surgeons when performing RLA. Since Zhang et al. [9] promoted and standardized lateral retroperitoneoscopic adrenalectomy (LRA), it has been the most commonly used method for treating adrenal tumors in China. In this position, we can rapidly and directly separate the adrenal tumor, which leads to a shorter operation time and less blood loss, as well as less postoperative complications. In our experience, even though the retroperitoneal approach is difficult, it is beneficial to the postoperative recovery of patients. Since the improvement of laparoscopic instruments and proficiency of operation skill, the tumor diameter may not be a major restricted factor [10] even though scholars have reported the successful use of the partial resection method in large adrenal tumors [11]. However, the opinions on the application of laparoscopy in the management of large (> 5 cm) adrenal tumors are controversial [12, 13].

In the present study, we performed a comprehensive analysis of minimally invasive surgery methods used in the management of large adrenal tumors, which included perioperative and long-term follow-up results. The results revealed that LRA was superior to TLA in terms of operation time, hospitalization duration, time to first oral intake and time to ambulation. Further analyses focusing on RLA for tumors with different diameters demonstrated that the intraoperative indicators in larger adrenal tumors (> 5 cm) showed superior results to those of smaller adrenal tumors (< 5 cm), while the postoperative indicators between the two groups showed similar results. On the other hand, we further analyzed the different resection methods in the RLA group. The results confirmed that the partial resection method was superior to the total resection method from the perspective of hormone supplementation. There were three patients in the RLA group and 2 patients in the TLA group who were converted to the ICU. Among these patients, four of them had pheochromocytoma (PHEO) with unstable blood pressure after the operation, which could be potentially caused by the over-secretion of catecholamines during the tumor disturbance [14]. Abnormal blood vessels and huge blood volumes were found in all of the PHEO patients. Both of these features could lead to sharp fluctuations in the blood pressure and heart rate of patients, as well as to the increase in bleeding.

The complication rate of RLA was usually approximately 11.5%, and the complications include adrenal cataclastic, peritoneal injury, vena cava injury and renal vein injury [15]. In addition, hypercapnia and pneumoderm, which are caused by the high pressure of CO_2_ or by a shallow insertion of the trocar, can also occur [16]. According to our experience, the CO_2_ insufflation pressure was usually between 12 cmH_2_O and 15 cmH_2_O. Studies have shown that these complications are mainly related to the different learning curves among surgeons [17, 18].

The inevitable bleeding from RLA will be less severe with only slight tissue injury when a small incision is made. Our experience suggested that we can inject approximately 350 ml normal saline or air into a homemade gasbag to expand the potential peritoneal cavity. Three minutes of compression was needed following this in order to prevent the bleeding caused by the rupture of small blood vessels. In addition, an ultrasonic knife could be used in the solidification of small blood vessels during the separation of the renal fascia and adipose capsule. However, open surgery hemostasis would be chosen without any hesitation when the operating vision was influenced by adrenal cataclastic or unmanageable vascular injury [19].

There were certain limitations of the RLA procedure that need to be taken into consideration. First, the restricted potential cavity has limited the diameter of the removable portion of the tumor. The interaction of surgical instruments could also affect the ease of the operation. Many scholars have reported that retroperitoneal laparoscopy could just be used for small-sized to medium-sized benign adrenal tumors [20]. However, the evidence from our present study has extended this restriction. Secondly, the normal anatomic marks of the peritoneal cavity would be disturbed by the use of a gasbag, which was usually used to expand the peritoneal cavity. This method may also enhance the difficulty of the operation. Third, the effect of gasbag extrusion could also make the tumor over-secrete catecholamines in PHEO patients; therefore, the preoperative control of blood pressure and sphygmus is very important [21].

It is well-known that there are potential limitations and biases of a retrospective analysis design that may affect the results. On one hand, the number of patients in the groups in the *RLA* vs. *TLA* and *partial resection* vs. *total resection of RLA* comparisons were not equal, which could lead to a potential bias of the results. However, we analyzed the baseline characteristics and measurements between the groups in this study. The results confirmed that there were no statistically significant differences in the basic characteristics between the groups, which could be used in further analyses of outcome indicators. On the other hand, the further subgroups analyses, which included the variables of obesity and adhesion, were not completed due to a lack of sufficient data. Moreover, due to the lack of a sufficient number of patients with malignant tumors, we cannot further perform a survival analysis to determine the overall survival and progression-free survival of adrenocortical carcinoma patients during the follow-up period.

## Conclusions

Based on our analyses, both RLA and TLA seem to provide similar effects for the surgical management of large adrenal tumors. However, for benign tumors, partial RLA should be considered to reduce the need for hormone supplementation. Our results have strengthened the opinion that RLA is an efficacious surgical intervention for the treatment of adrenal tumors larger than 5 cm in diameter.

## Additional file

Additional file 1 STROBE Statement checklist of obserational studies. (DOCX 42 kb)

## Abbreviations

BMI: Body mass index
CR: Complete remission
EBL: Estimate blood loss
ICU: Intensive care unit
OT: Operation time
RFR: Recurrence-free rate
RLA: Retroperitoneal laparoscopic adrenalectomy
TLA: Transperitoneal laparoscopic adrenalectomy
